# SRC-2 Regulates Brain Endothelial and Neuronal Metabolism in a Mouse Model of Neurodegeneration

**DOI:** 10.3390/ijms27219943

**Published:** 2026-11-06

**Authors:** Rohit K. Srivastava, Pratibha Singh, Emir S. Jonsson, Irfana Saleem, Caycel J. P. Desales, David M. Lonard

**Affiliations:** Department of Molecular and Cellular Biology, Baylor College of Medicine, Houston, TX 77030, USA; pratibha.singh@bcm.edu (P.S.); emir.jonsson@bcm.edu (E.S.J.); irfana.saleem@bcm.edu (I.S.); cayceljohn.desales@bcm.edu (C.J.P.D.)

**Keywords:** neurodegeneration, brain, steroid receptor coactivators

## Abstract

Cerebrovascular dysfunction and impaired brain energy metabolism are increasingly recognized as important contributors to neurodegenerative diseases, but their molecular regulators remain poorly understood. Here, we examined the roles of steroid receptor coactivator-2 (SRC-2/*NCOA2*) in the cerebrovascular and metabolic changes associated with neurodegeneration using ps19 mice as a model. In our study, we found a significant decrease in cortical SRC-2 expression in ps19 mice compared with wild-type (WT) controls, with no changes in SRC-1 and SRC-3 levels between the two groups. ps19 mice showed reduced cortical endothelial and blood–brain barrier-associated markers (CD31, occludin, claudin-5, total eNOS (endothelial nitric oxide synthase), and eNOS Ser1177) and increased eNOS Thr495 phosphorylation. HIF-1α, *GLUT2*, and *GLUT4* were also reduced in ps19 mice, whereas *GLUT1* and *GLUT3* were unchanged between the groups. To examine the role of SRC-2, we used siRNA to reduce its expression in brain endothelial cells and primary neurons. In brain endothelial cells, SRC-2 knockdown reduced the expression of endothelial and BBB-associated markers, including *CD31* and *occludin*, as well as *GLUT3*, while increasing inhibitory eNOS Thr495 phosphorylation and *GLUT4* expression. In brain endothelial cells, SRC-2 knockdown significantly reduced the extracellular acidification rate (ECAR) and NAD^+^/NADH ratio. Knockdown of SRC-2 in primary neurons (from WT and ps19 mice) significantly reduced the oxygen consumption rate (OCR), while the NAD^+^/NADH ratio remained unchanged. These findings identify SRC-2 as a regulator of endothelial and neuronal metabolism and support further investigation of SRC-2-dependent pathways as potential therapeutic targets in neurodegenerative diseases.

## 1. Introduction

Vascular abnormalities, including reduced cerebral blood flow, blood–brain barrier (BBB) disruption, and dysregulated neurovascular coupling, are being increasingly recognized as key contributors to the progression of Alzheimer’s disease (AD) and related neurodegenerative disorders, as such abnormalities may disrupt neurovascular unit function and impair the delivery of oxygen and glucose to neurons, which are needed for neuronal activity and energy production [1,2,3]. The brain has a high and continuous demand for oxygen and glucose; therefore, the maintenance of neuronal function depends on tight regulation of the interactions between the cerebral vasculature and brain energy metabolism [4,5,6].

Brain endothelial cells form the BBB and control the transport of nutrients, metabolites, and signaling molecules between the circulation and central nervous systems [7,8]. Glucose transport across the BBB is a critical step that links cerebral vascular function to neuronal energy metabolism [9]. *Glucose transporter 1* (*GLUT1*) is the major transporter responsible for glucose transport from the circulation to the brain and is highly expressed in brain endothelial cells [9], whereas *GLUT3* is mainly expressed in neurons and regulates neuronal glucose uptake. *GLUT2* and *GLUT4* are expressed only in certain types of cells in the central nervous system [9,10]. Decreased glucose transport has been suggested to be involved in the pathology of AD-related models, even when glucose is available. In particular, reduced *GLUT1* expression in endothelial cells has been associated with microvascular degeneration, reduced cerebral blood flow, breakdown of the BBB, impaired neuronal activity, and increased neurodegeneration [11,12]. BBB permeability may also be influenced by lipid metabolism in neurodegenerative diseases. Studies have shown that altered cholesterol and triglyceride levels are associated with BBB dysfunction [13,14], while APOE4 has been linked to increased BBB permeability in AD and other neurodegenerative disorders [15,16]. Together, these findings indicate that both glucose transporter expression and lipid-related changes in BBB integrity may influence nutrient transport and neuronal energy metabolism.

Beyond glucose transport itself, the delivery of oxygen and glucose through the cerebral vasculature is essential for maintaining neuronal energy metabolism [17]. Neuronal ATP is produced through both mitochondrial oxidative phosphorylation and glycolysis, and impaired delivery of oxygen or glucose can shift the balance between these pathways [18]. Changes in the availability of oxygen and nutrients induce adaptive programs, which can affect cellular energy metabolism [19]. The main transcriptional regulator of this response is hypoxia-inducible factor-1α (HIF-1α), which regulates genes involved in glucose uptake, glycolysis, angiogenesis, and vascular adaptation [20]. Reduced oxygen availability in endothelial cells promotes increased dependence on glucose utilization and glycolysis and decreased mitochondrial respiration and oxidative metabolism [21]. Therefore, altered expression of HIF-1α could be a marker of vascular and metabolic stress in addition to tissue hypoxia. Because glucose transporters themselves are regulated by HIF-1α, glucose transport and hypoxic signaling in brain endothelial cells are closely linked, and identifying the transcriptional regulators that coordinate these pathways is important for understanding vascular and metabolic dysfunction in AD. Recent studies have highlighted the potential of targeting vascular and metabolic dysfunction in neurodegenerative diseases. Experimental and clinical studies suggest that improving cerebral glucose metabolism, BBB function, and mitochondrial activity may offer therapeutic benefits [11,15,22,23]. These findings support further investigation of the molecular regulators involved in brain vascular function and energy metabolism.

One group of transcriptional regulators that could connect these pathways is the steroid receptor coactivator family. Steroid receptor coactivators (SRCs) are widely expressed in the mammalian brain and are involved in nuclear receptor-dependent pathways that control neuroendocrine regulation, behavior, learning, and memory [24,25]. The SRC family members, which include SRC-1/*NCOA1*, SRC-2/*NCOA2*, and SRC-3/*NCOA3*, are transcriptional coregulators that enhance gene expression through interactions with ligand-activated nuclear receptors and other transcription factors [26]. SRC proteins do not directly bind DNA but instead enhance transcription by recruiting chromatin-modifying enzymes and components of the basal transcriptional machinery [27]. SRCs regulate cellular responses to hormonal, metabolic, and environmental signals and have established roles in reproduction, immunity, metabolism, cardiovascular biology, and cancer [28,29].

Notably, SRC family members have been reported to interact with HIF-1α and to modulate hypoxia-responsive and metabolic gene expression [30], suggesting that they may influence glucose transport and hypoxic adaptation in brain endothelial cells. However, their roles in the BBB and in AD-related vascular and metabolic dysfunction remain poorly understood.

Most studies of SRCs in the brain have focused on neuronal function rather than the cerebrovasculature. SRC-1 has been implicated in steroid hormone signaling, synaptic plasticity, learning, and memory [25], while SRC-3 has been shown to regulate hippocampal synaptic plasticity and memory formation [31].

In contrast, little is known about SRC-2’s role in neurodegenerative disease and brain homeostasis, though it is known to be expressed in several brain regions and the pituitary gland [25,32,33,34]. Our initial analysis showed that SRC-2 expression was higher than those of SRC-1 and SRC-3 in the mouse cerebral cortex and was significantly reduced in ps19 mice compared with wild-type (WT) controls. These observations, together with the established role of SRC-2 in systemic glucose and lipid metabolism, circadian metabolic regulation, and immune-cell differentiation [35,36,37,38], provided the rationale for investigating its potential involvement in tau-associated neurodegeneration. Whether SRC-2 contributes to cerebrovascular dysfunction and metabolic dysregulation in neurodegeneration has not been examined.

In the present study, we used ps19 mice, which express human P301S mutant tau and develop progressive tau pathology and neurodegeneration, as a model of tauopathy relevant to the tau-associated neurodegenerative component of AD [39], to investigate the potential role of SRC-2 in tau-associated cerebrovascular and metabolic dysfunction. We hypothesized that SRC-2 plays an important role in maintaining brain endothelial function and neuronal energy metabolism, and that reduced SRC-2 expression may contribute to vascular and metabolic changes associated with tauopathy.

We examined cortical SRC-2 expression in ps19 mice compared to WT mice and investigated the expression of endothelial markers, HIF-1α, and *GLUT1–4*.

To further investigate the functional significance of SRC-2 in brain endothelial cells, we used siRNA-mediated SRC-2 knockdown to evaluate its role in regulating endothelial and tight junction markers, endothelial nitric oxide synthase (eNOS) signaling, and nitric oxide production. We also investigated cellular bioenergetic function by measuring oxygen consumption rate (OCR) and extracellular acidification rate (ECAR) in brain endothelial cells as well as neurons from WT and ps19 mice. Together, these findings provide insight into the potential involvement of SRC-2 in cerebrovascular and neuronal metabolic dysfunction associated with neurodegeneration. Our results suggest that SRC-2 is involved in maintaining the vascular and neuronal metabolic pathways that are impaired in ps19 mouse model of neurodegeneration.

## 2. Results

### 2.1. SRC-2 Expression Is Reduced in the Cortex of ps19 Mice

**Figure 1:**
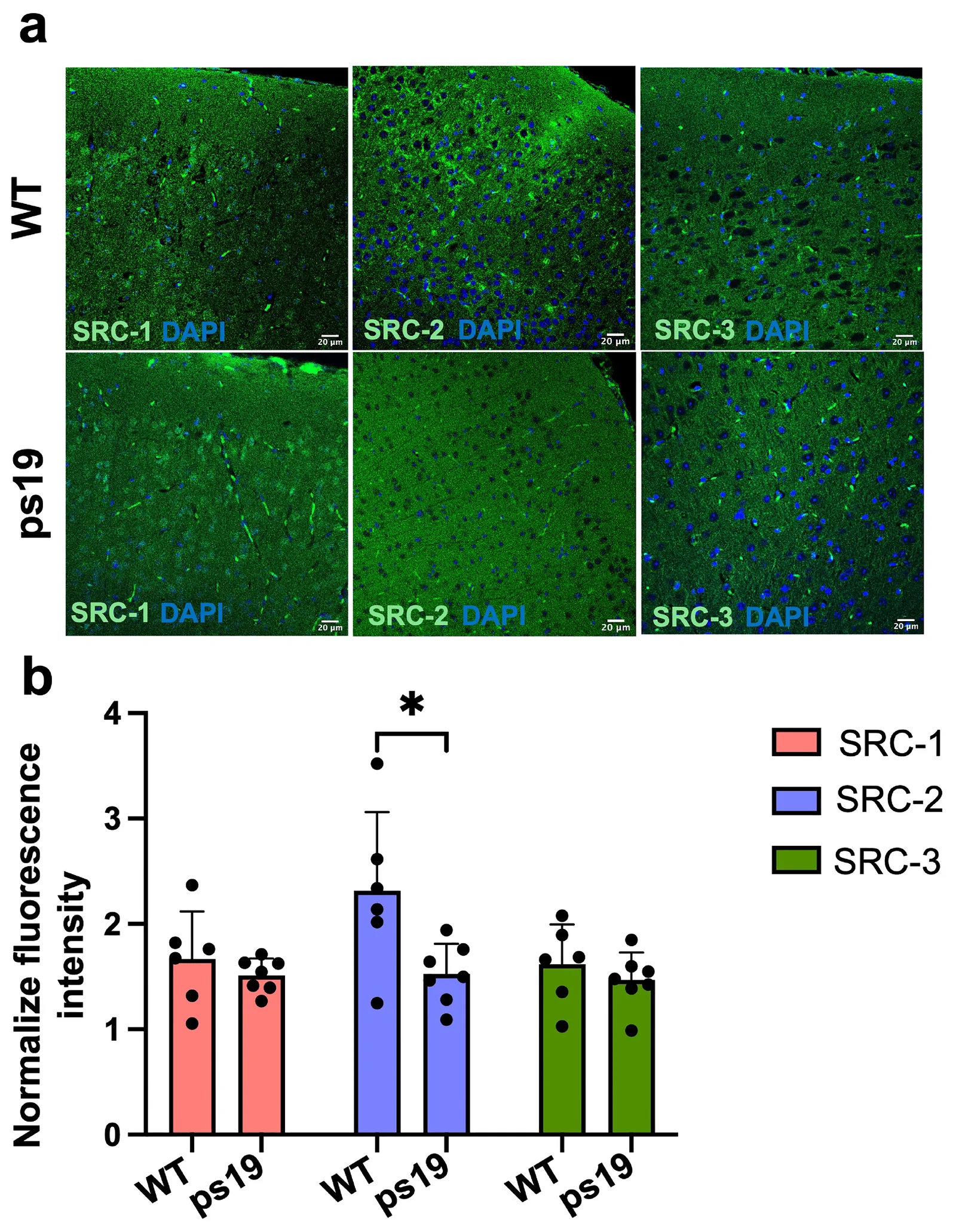
Reduced SRC-2 expression in the cerebral cortex of ps19 mice. (**a**) Representative immunofluorescence images showing SRC-1, SRC-2, and SRC-3 expression (green) in the cerebral cortex of wild-type (WT, *n* = 5) and ps19 mice (*n* = 6). Nuclei were counterstained with DAPI (blue). (**b**) Quantification of fluorescence intensity showed significantly reduced SRC-2 expression in ps19 mice compared with WT controls, whereas SRC-1 and SRC-3 expression remained unchanged. * *p* < 0.05.

To examine if the expressions of SRC-1, SRC-2 and SRC-3 are altered in the ps19 model of tauopathy, we measured their levels in the cortex of ps19 mice and non-transgenic littermate (WT) controls using immunofluorescence. Among the three SRC family members, SRC-2 showed the highest expression in cortical tissue (Figure 1a). Quantification showed a substantial decrease in SRC-2 levels in ps19 mice compared with WT controls. Meanwhile, there was no significant difference in SRC-1 and SRC-3 expression between the two groups (Figure 1b). Based on these findings, we focused our subsequent studies on SRC-2 to explore its potential role in tau-associated cerebrovascular and metabolic dysfunction.

### 2.2. SRC-2 Knockdown Reduces Endothelial and Blood–Brain Barrier-Associated Markers in Brain Endothelial Cells

**Figure 2:**
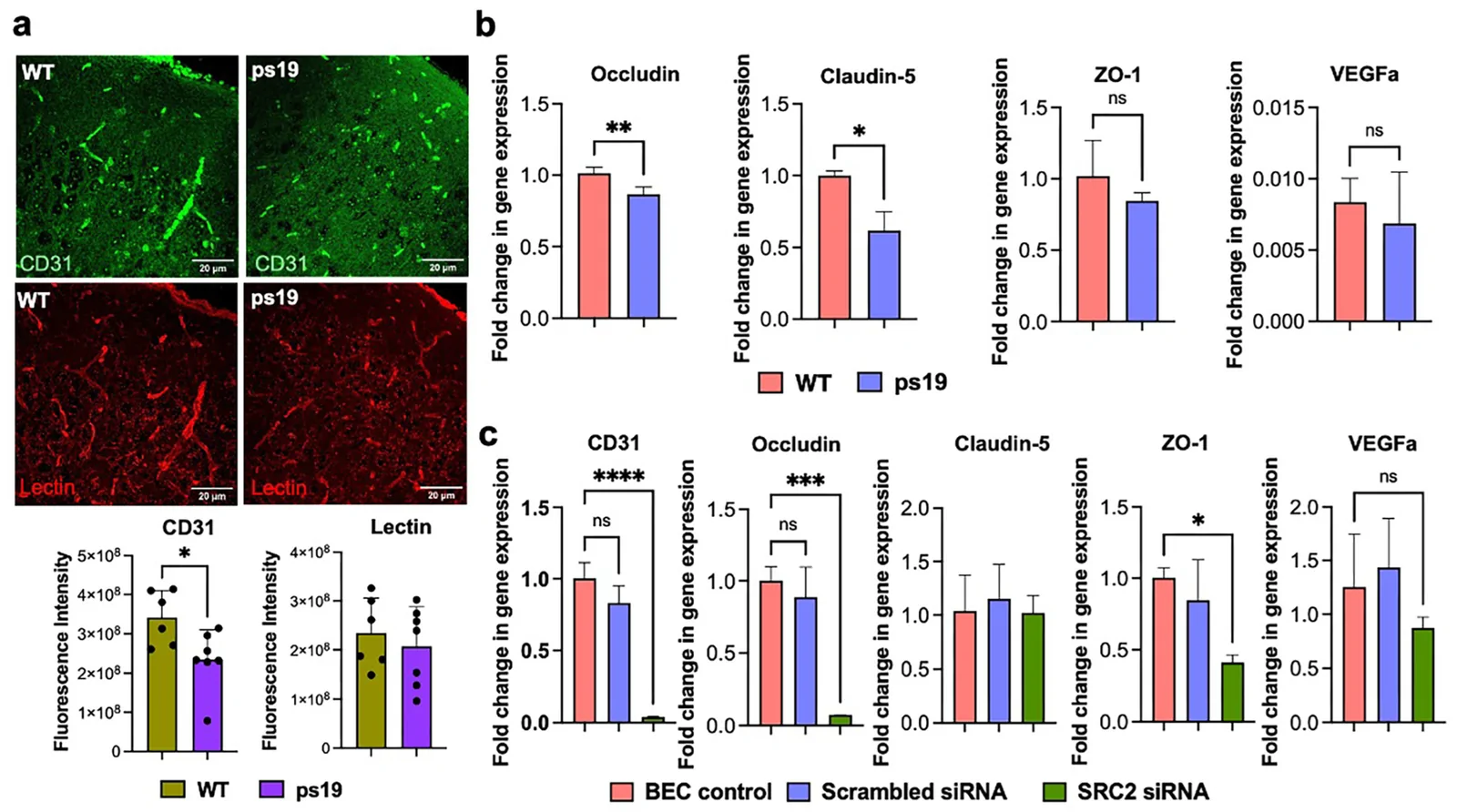
Alterations in endothelial and blood–brain barrier-associated markers in ps19 mice and SRC-2 knockdown effect on brain endothelial cells. (**a**) Representative immunofluorescence images showing CD31 (green) and lectin (red) staining in the cerebral cortex of WT (*n* = 5) and ps19 mice (*n* = 7). Quantification of fluorescence intensity showed significantly reduced CD31 expression in ps19 mice, whereas lectin staining remained unchanged. (**b**) Quantitative real-time PCR (qPCR) analysis of Occludin, claudin-5, ZO-1, and VEGFa expressions in cortical tissue from WT and ps19 mice. (**c**) qPCR analysis of CD31, occludin, claudin-5, ZO-1, and VEGFa expressions in brain endothelial cells following siRNA-mediated SRC-2 knockdown compared with untreated and scrambled siRNA controls. * *p* < 0.05, ** *p* < 0.01, *** *p* < 0.001, **** *p* < 0.0001; ns, not significant.

Next, we examined the brain endothelium in the ps19 mouse cortex. Immunofluorescence analysis showed a significant decrease in CD31 expression, but not lectin levels, in ps19 mice compared to WT controls (Figure 2a). We then performed qPCR to evaluate the expressions of endothelial- and BBB-associated genes in cortical tissue. ps19 mice showed a significant decrease in the expressions of *Occludin* and *claudin-5* compared to non-transgenic controls. However, there was no significant difference in the expression of *ZO-1* or *VEGFa* between the two groups (Figure 2b). To further investigate the role of SRC-2 in brain endothelial function, we knocked down SRC-2 in cultured brain endothelial cells using siRNA and measured the expressions of *CD31*, *VEGFa*, *occludin*, *claudin-5*, and *ZO-1*. Knockdown of SRC-2 caused a significant decrease in the expressions of *CD31*, *occludin* and ZO-1 compared to control cells. SRC-2 knockdown also reduced *VEGFa level*, but this was not significant. SRC-2 knockdown did not change the expression of *claudin-5* (Figure 2c).

### 2.3. SRC-2 Knockdown Increases Inhibitory eNOS Thr495 Phosphorylation in Brain Endothelial Cells

**Figure 3:**
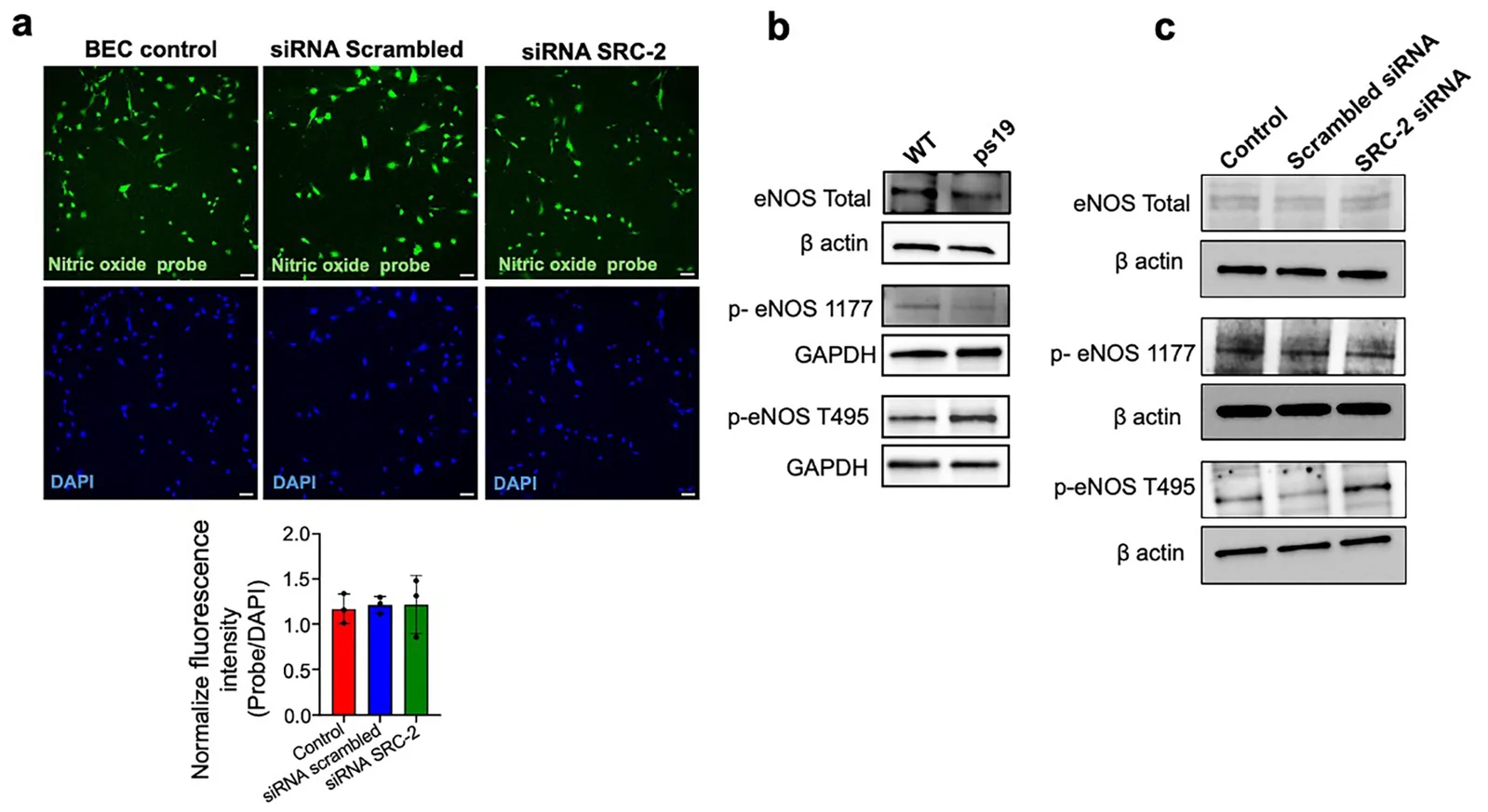
SRC-2 knockdown alters endothelial nitric oxide synthase (eNOS) phosphorylation in brain endothelial cells. (**a**) Representative immunofluorescence images showing nitric oxide production in untreated control, scrambled siRNA-treated, and SRC-2 siRNA-treated brain endothelial cells using a nitric oxide-sensitive fluorescent probe (green). Nuclei were counterstained with DAPI (blue). Quantification of fluorescence intensity showed no significant differences in nitric oxide levels among the groups. The scale bars in figure corresponds to 40 µm. (**b**) Western blots showing total eNOS, phosphorylated eNOS at Ser1177 (p-eNOS Ser1177), and phosphorylated eNOS at Thr495 (p-eNOS Thr495) in cortical tissue from WT and ps19 mice. β-actin was used as a loading control for total eNOS, while GAPDH was used as a loading control for p-eNOS Ser1177 and p-eNOS Thr495. (**c**) Western blots showing total eNOS, p-eNOS Ser1177, and p-eNOS Thr495 in untreated control, scrambled siRNA-treated, and SRC-2 siRNA-treated brain endothelial cells. β-actin was used as a loading control for all.

To further study SRC-2s linked to vascular function, we investigated endothelial nitric oxide signaling. Nitric oxide is an important regulator of vascular tone and cerebral blood flow and plays a role in endothelial and BBB homeostasis. First, we determined the nitric oxide levels in brain endothelial cells following SRC-2 knockdown (using a probe). Nitric oxide levels were similar in control and SRC-2 siRNA-treated cells, showing that SRC-2 knockdown did not change basal nitric oxide levels (Figure 3a). Since measuring nitric oxide levels in mice was not feasible, we next examined eNOS signaling, which regulates nitric oxide production [40], in the cortex of ps19 mice and WT controls. eNOS activity is regulated by phosphorylation at activating and inhibitory sites, and we measured total eNOS levels, phosphorylation at Ser1177 (activating site), and phosphorylation at Thr495 (inhibitory site). The Western blot results showed that the total eNOS and p-eNOS Ser1177 levels were higher in the cortex of WT than in ps19 mice. In contrast, p-eNOS Thr495 was elevated in the ps19 cortex (Figure 3b).

To determine whether SRC-2 mediates these changes, we examined the same eNOS markers in brain endothelial cells after SRC-2 knockdown. Total eNOS and p-eNOS Ser1177 levels were not different between SRC-2 knockdown cells and control cells. However, p-eNOS Thr495 was upregulated in SRC-2 knockdown cells. This increase was similar to what was observed in ps19 cortices (Figure 3c). Altogether, these results show that ps19 mice have different levels of eNOS phosphorylation, with reduced activating and elevated inhibitory phosphorylation. Knockdown of SRC-2 caused an increase in inhibitory Thr495 phosphorylation in brain endothelial cells, while total eNOS, Ser1177 phosphorylation, and basal NO levels were unchanged. Together, these findings indicate a role for SRC-2 in regulating endothelial eNOS phosphorylation and suggest that SRC-2 contributes to maintaining eNOS signaling by limiting inhibitory phosphorylation at Thr495.

### 2.4. SRC-2 Knockdown Differentially Regulates Glucose Transporter Expression in Brain Endothelial Cells

**Figure 4:**
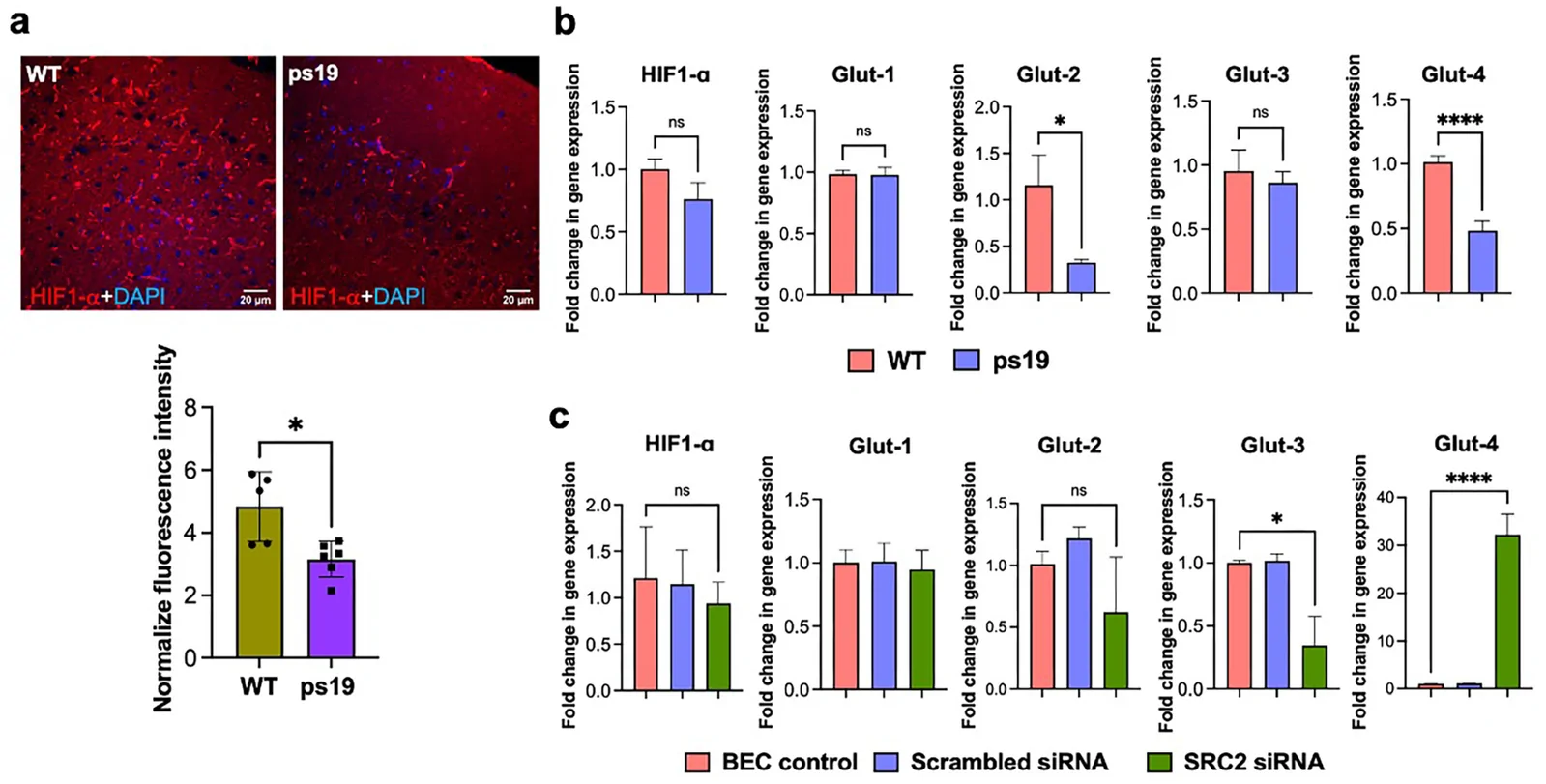
Altered HIF-1α and glucose transporter expressions in ps19 cortex and following SRC-2 knockdown in brain endothelial cells. (**a**) Representative immunofluorescence images showing HIF-1α expression (red) in the cerebral cortex of WT (*n* = 5) and ps19 mice (*n* = 6). Nuclei were counterstained with DAPI (blue). Quantification of fluorescence intensity showed decreased HIF-1α expression in ps19 mice compared with WT controls. (**b**) qPCR analysis of HIF-1α, *GLUT1, GLUT2, GLUT3*, and *GLUT4* expressions in cortical tissue from WT and ps19 mice. (**c**) qPCR analysis of HIF-1α, *GLUT1, GLUT2, GLUT3*, and *GLUT4* expressions in untreated control, scrambled siRNA-treated, and SRC-2 siRNA-treated brain endothelial cells. * *p* < 0.05, **** *p* < 0.0001; ns, not significant.

Next, we measured the expressions of HIF1α and glucose transporters in the cortex of 9-month-old ps19 and WT mice. HIF1α expression was significantly lower in ps19 compared with WT mice, suggesting that the ability to increase HIF1α expression in response to metabolic stress may be reduced in the cortex of ps19 mice (Figure 4a). Because HIF-1α is an important regulator of glucose metabolism, we examined *GLUT1–4* expression in the same cortical tissue to assess glucose transporter changes in ps19 mice. *GLUT1* and *GLUT3* expressions were similar between ps19 and WT mice, while expressions of *GLUT2* and *GLUT4* were significantly reduced in ps19 mice (Figure 4b). To determine whether SRC-2 regulates HIF-1α and glucose transporter expressions in brain endothelial cells, we measured their expressions in brain endothelial cells following SRC-2 knockdown. SRC-2 knockdown did not alter HIF1α expression, although we see a decreasing trend in HIF1α in SRC-2 knockdown brain endothelial cells but result is non-significant. *GLUT1* remained unchanged, while *GLUT2* (non-significant) and *GLUT3* (significant) expressions decreased following SRC-2 knockdown. *GLUT4* showed increased expression after SRC-2 knockdown compared to control cells, although the reason for this increase is not clear (Figure 4c). These data suggest that SRC-2 may have a protective role at the brain endothelium by supporting *GLUT2* and *GLUT3* expression, while the changes in *GLUT4* and HIF1α observed in ps19 mice are likely to involve mechanisms independent of endothelial SRC-2.

### 2.5. SRC-2 Knockdown Impairs Cellular Bioenergetics and Reduces the NAD^+^/NADH Ratio in Brain Endothelial Cells

Mitochondrial function was assessed by measuring the OCR of primary neurons from WT and ps19 mice that were either left untreated or treated with scrambled siRNA or SRC-2 siRNA (Supplementary Figure S1a,b). WT control neurons showed a higher basal OCR than ps19 control neurons. Interestingly, scrambled siRNA treatment increased OCR in both WT and ps19 neurons compared with controls. SRC-2 knockdown significantly reduced OCR in WT neurons compared with scrambled-siRNA-treated WT neurons (Supplementary Figure S1a–c). In ps19 neurons, after SRC-2 knockdown, the OCR was similar to that of the untreated ps19 control but was significantly lower than the ps19 scrambled-siRNA group (Supplementary Figure S1b,c). Because scrambled siRNA itself increased OCR, scrambled-siRNA-treated neurons were used as the main control for evaluating the effect of SRC-2 knockdown in order to observe the change in mitochondrial activity due solely to SRC-2 knockdown.

**Figure 5:**
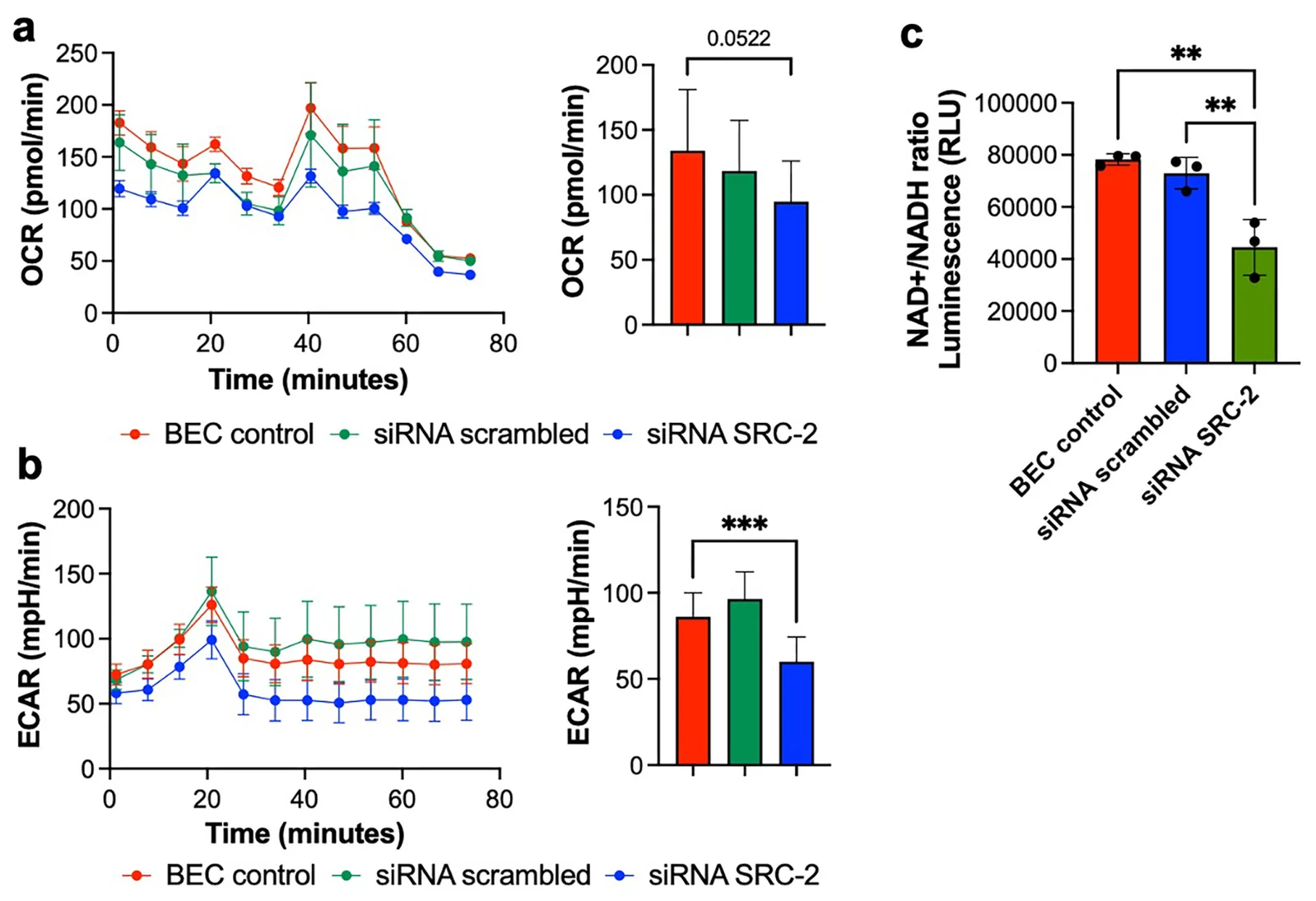
SRC-2 knockdown alters cellular bioenergetics and reduces the NAD^+^/NADH ratio in brain endothelial cells. (**a**) Oxygen consumption rate (OCR) in untreated control, scrambled siRNA-treated, and SRC-2 siRNA-treated brain endothelial cells, measured using the Seahorse extracellular flux analyzer. The line graph shows OCR over time, and the bar graph represents the corresponding OCR quantification. (**b**) Extracellular acidification rate (ECAR) in the same experimental groups, with the line graph showing ECAR over time and the bar graph representing the corresponding ECAR quantification. (**c**) NAD^+^/NADH ratio measured in untreated control, scrambled siRNA-treated, and SRC-2 siRNA-treated brain endothelial cells. ** *p* < 0.01, *** *p* < 0.001. In both the line graphs and corresponding bar graphs of figure (**a**,**b**), red represents untreated control cells, green represents scrambled siRNA-treated cells, and blue represents SRC-2 siRNA-treated cells.

Interestingly, a clear decrease in OCR after oligomycin treatment was not observed in any neuronal groups (Supplementary Figure S1a,b). Since the oligomycin response is used to calculate ATP-linked respiration and proton leak, these values should be interpreted with caution because oligomycin did not produce the expected decrease in OCR. Another study also reported a weak oligomycin response in which the OCR in PINK1-mutant neural cells did not decrease after oligomycin treatment, but they remained responsive to FCCP and rotenone [41]. Overall, SRC-2 knockdown reduced mitochondrial respiration, especially when compared with scrambled-siRNA controls. We next examined the effect of SRC-2 knockdown on metabolism in brain endothelial cells. SRC-2 knockdown resulted in a lower OCR compared with scrambled-siRNA-treated brain endothelial cells, though the difference was not significant (Figure 5a). In contrast, ECAR was significantly reduced after SRC-2 knockdown, indicating decreased glycolytic activity. These results suggest that knockdown of SRC-2 changes metabolic function in brain endothelial cells, with a clear effect on glycolysis and a trend toward reduced mitochondrial respiration (Figure 5b). We also measured the NAD^+^/NADH ratio in both neurons and brain endothelial cells. The NAD^+^/NADH ratio was lower in ps19 neurons than in WT neurons, suggesting reduced oxidation of NADH and impaired redox balance in ps19 neurons. SRC-2 knockdown did not change the NAD^+^/NADH ratio in WT or ps19 neurons (Supplementary Figure S2). In brain endothelial cells, however, SRC-2 knockdown significantly reduced the NAD^+^/NADH ratio compared with scrambled-siRNA controls, indicating a shift toward a more reduced cellular redox state (Figure 5c). Overall, these results show that SRC-2 contributes to the maintenance of cellular metabolism. In brain endothelial cells, SRC-2 knockdown reduced ECAR, significantly decreased the NAD^+^/NADH ratio, and showed a trend toward a lower OCR. The neuronal studies further support a role for SRC-2 in maintaining mitochondrial respiration and show that ps19 neurons have altered mitochondrial respiration and NAD redox balance.

## 3. Discussion

In the present study, we examined the role of SRC-2 in the vascular and metabolic changes in ps19 mice, a well-established mouse model for neurodegeneration. The major finding was that SRC-2 expression was decreased in the cortex of ps19 mice compared to WT, while SRC-1 and SRC-3 were unchanged between the two groups. Following this decrease, changes in endothelial and BBB-associated markers, eNOS phosphorylation, HIF-1α and glucose transporter gene expressions, and neuronal mitochondrial respiration were observed. SRC-2 knockdown experiments in brain endothelial cells helped establish its role in vascular function, while Seahorse experiments in brain endothelial cells and neurons provided further evidence of its involvement in cellular metabolism. SRC-2 knockdown did not reproduce all of the changes, which is expected as neurodegeneration involves multiple pathways and regulatory mechanisms. As ps19 mice were used as a model of neurodegeneration in this study, these findings suggest that reduced SRC-2 expression may contribute to the vascular and metabolic dysfunction associated with neurodegeneration. The involvement of SRC-2 in other neurological disorders has been investigated, particularly in experimental autoimmune encephalomyelitis (EAE), a mouse model of multiple sclerosis. Mimouna et al. demonstrated that deletion of SRC-2/GRIP1 in myeloid cells reduced neuroinflammation, microglial activation, and demyelination during the neuroinflammatory phase of EAE [42]. In contrast, Zhang et al. reported that SRC-2 deficiency in regulatory T cells impaired their differentiation and increased EAE severity [43]. Another study demonstrated that T-cell-specific SRC-2 deletion impaired CD4^+^ T-cell activation and reduced EAE severity [37]. These findings indicate that SRC-2 can have different effects depending on the cell type involved. However, its role in neurodegenerative diseases such as Alzheimer’s and Parkinson’s diseases remains poorly understood. In our study, SRC-2 expression was reduced in the cortex of 9-month-old ps19 mice, suggesting its potential involvement in tau-associated neurodegeneration. Since our analysis was limited to a single age, we cannot determine whether SRC-2 expression changes during earlier stages of tau pathology or how its role may differ as the disease progresses. Further studies examining SRC-2 expression and function at different disease stages will be necessary to establish its contribution to neurodegenerative disease progression.

Our vascular findings showed a significant reduction in CD31 immunofluorescence in the cortex of ps19 mice compared with WT controls, while lectin-positive vascular density remained unchanged. Similarly, the expression levels of *occludin* and *claudin-5* were significantly decreased in ps19 mice, whereas *ZO-1* and *VEGFa* showed a non-significant reduction. The unchanged lectin signal is important because it shows that the lower CD31 signal does not simply indicate fewer blood vessels. CD31 is an endothelial adhesion and signaling protein, whereas lectin binds to carbohydrate structures on the vascular endothelium [44,45]. Therefore, changes in CD31 expression or distribution may reduce CD31 staining without corresponding changes in lectin labeling. CD31 also participates in endothelial responses to blood flow. Tzima et al. demonstrated that CD31 forms a signaling complex with VE-cadherin and VEGFR2 (Vascular Endothelial Growth Factor Receptor 2) that regulates endothelial responses to mechanical forces [46].

A similar finding was reported by Hussong et al. (2023) in ps19 mice, where cortical lectin-positive vascular density did not change despite a severe impairment of endothelium-dependent cerebral blood flow responses [47]. Similarly, Park et al. (2020) reported modest reductions in resting cerebral blood flow in the entorhinal cortex and hippocampus and impaired neurovascular responses in young ps19 mice [48]. In our study, SRC-2 knockdown reduced CD31 expression in cultured brain endothelial cells, suggesting that SRC-2 may contribute to the regulation of this endothelial marker. Together, these findings suggest that changes in endothelial CD31 expression and vascular function can occur without a corresponding reduction in lectin-positive vascular labeling.

The reduced expression of *occludin* and *claudin-5* also suggests impaired tight-junction control in the ps19 cortex. Both proteins are important components of endothelial tight junctions that maintain the BBB [49,50]. Their decreased expression means that the integrity of tight junctions is impaired in ps19 mice. However, permeability was not directly measured in this study, and these data alone cannot prove that the BBB is leaky. Whole cortical tissue was used to measure *occludin* and *claudin-5* levels, so we cannot determine if brain endothelial cells specifically had reduced expression of these genes. These findings are consistent with those of Guzmán-Hernández and Fossati, who showed that aggregated tau led to the breakdown of endothelial junction proteins and increased BBB permeability in cultured cerebral endothelial cells. They also found abnormal junction-protein localization and other vascular changes in 3-month-old P301S mice [51].

The SRC-2 knockdown experiments provide further evidence that SRC-2 contributes to these endothelial changes. SRC-2 knockdown in brain endothelial cells resulted in decreased levels of CD31, *occludin* and *ZO-1. VEGFa* also showed a downward trend but was non-significant. However, *claudin-5* did not change after SRC-2 knockdown. This does not argue against a role for SRC-2, since reducing one regulator in isolated endothelial cells would not be expected to reproduce every change present in the ps19 brain. The ps19 cortex contains multiple cell types and is exposed to chronic tau pathology and inflammatory signals [52]. In addition, the pathogenic tau itself may directly affect cerebral endothelial cells [51]. Hussong et al. (2023) demonstrated that soluble pathogenic tau enters brain microvascular endothelial cells and impairs vascular function [47], and Guzmán-Hernández and Fossati (2025) showed that fibrillar tau alters endothelial metabolism and barrier function [51]. Thus, our knockdown data supports a role for SRC-2 in several of the endothelial changes found in ps19 mice, while other effects of tau pathology also contribute to the vascular phenotype.

The eNOS findings provide another link between SRC-2 and vascular dysfunction in the ps19 mice. Overall eNOS levels and phosphorylation at the activating Ser1177 site were reduced in ps19 cortex, while phosphorylation at the inhibitory Thr495 site was increased. Together, these changes are consistent with decreased eNOS activation. Interestingly, Hussong et al. (2023) found a similar pattern in brain microvessels from ps19 mice, showing reduced activating eNOS phosphorylation and increased Thr495 phosphorylation [47]. These changes were followed by impaired endothelium-dependent cerebral blood flow responses, providing independent evidence that altered eNOS regulation contributes to the vascular phenotype in ps19 mice.

SRC-2 knockdown showed the same increase in inhibitory eNOS Thr495 phosphorylation that was observed in the ps19 cortex. In contrast, total eNOS and Ser1177 phosphorylation remained unchanged after SRC-2 knockdown, and basal nitric oxide levels were also not altered. These findings suggest that SRC-2 contributes to eNOS regulation, particularly at the Thr495 site. The reductions in total eNOS and Ser1177 phosphorylation in ps19 mice are therefore likely influenced by additional mechanisms present in the disease model. The unchanged basal nitric oxide level after SRC-2 knockdown also shows that increased Thr495 phosphorylation alone did not lead to a measurable decrease in nitric oxide under the conditions tested.

HIF-1α was also reduced at both the protein and mRNA levels in the cortex of ps19 mice. This result is important because HIF-1α is normally stabilized when oxygen availability decreases and can increase the expression of genes involved in glycolysis and glucose utilization [53]. Therefore, a decrease in HIF-1α in the ps19 cortex should not be considered an indicator of hypoxia. Instead, the data show that HIF-1α regulation is altered in the ps19 brain. Direct measurements of tissue oxygenation would be required to know whether this model shows reduced oxygen availability.

The changes in expression of the different glucose transporters varied in our study. *GLUT2–4* levels decreased (the decrease in *GLUT3* was non-significant) in ps19 cortices, whereas *GLUT1* level was unchanged. This difference is important because *GLUT1* is the major glucose transporter at the BBB [54]. Previous work has shown that reducing endothelial *GLUT1* levels can cause cerebral microvascular dysfunction, reduced blood flow, and BBB breakdown in an amyloid mouse model [11]. In our ps19 mice, however, *GLUT1* expression did not decrease. Therefore, the observed vascular changes were not associated with changes in overall cortical *GLUT1* expression. The significant reductions in *GLUT2* and *GLUT4* show that the expression of some glucose transporters is altered in ps19 cortices, although whole-cortex measurements cannot determine if these changes only occur in specific cells and which types.

SRC-2 knockdown also changed glucose transporter expression in brain endothelial cells, although the pattern was different from that observed in ps19 cortices. *GLUT2* and *GLUT3* decreased after SRC-2 knockdown, whereas *GLUT4* increased and *GLUT1* remained unchanged. HIF-1α was also unchanged. These results indicate that SRC-2 can regulate glucose transporter expression in endothelial cells, but they do not support a simple pathway in which SRC-2 controls these transporters through HIF-1α. The cultured endothelial cell experiment examined the effect of SRC-2 knockdown in a single cell type, whereas the cortex measurements reflect signals from many different cell types and are also affected by tau pathology. The two experiments, therefore, address related but different parts of the metabolic changes occurring in the ps19 brain.

The neuronal bioenergetics results provide functional support for the role of SRC-2 in cellular energy metabolism. ps19 neurons had a lower basal OCR than WT neurons, consistent with previous reports of mitochondrial abnormalities in P301S tauopathy. López-González et al. (2015) reported altered mitochondrial function during disease progression in ps19 mice [55], and recent work by Estrella et al. showed mitochondrial energetic abnormalities in the P301S model [56]. In our study, SRC-2 knockdown reduced OCR compared to the corresponding scrambled-siRNA controls, showing that knockdown of SRC-2 can impair neuronal mitochondrial respiration.

A role for SRC-2 in mitochondrial metabolism has also been reported in other cell types. Zhong et al. (2023) showed that loss of *NCOA2*/SRC-2 in CD8^+^ T cells reduced mitochondrial respiration and impaired oxidative phosphorylation [57]. Although T cells and neurons have different metabolic requirements, this study provides independent evidence that SRC-2 can regulate mitochondrial function. Our neuronal data extend this observation by showing that SRC-2 knockdown also leads to decreased mitochondrial respiration in neurons.

The Seahorse experiments on neurons isolated from mice also showed that scrambled-siRNA treatment increased OCR compared with control cells. Lipid-based transfection reagents have previously been shown to alter neuronal bioenergetics and mitochondrial function [58]. Therefore, the best comparison for evaluating SRC-2 knockdown is with the scrambled-siRNA control because both groups underwent the same transfection procedure. However, we observed a weak response to oligomycin, although this was also reported by a previous study [41]. This limits the interpretation of the ATP-linked respiration and proton leak results, but it does not prevent a comparison of the basal OCR between the matched scrambled and SRC-2 knockdown groups.

The experiments on brain endothelial cells also support a role for SRC-2 in cell metabolism. SRC-2 knockdown significantly reduced ECAR and caused a small decrease in OCR that was not statistically significant. Brain endothelial cells depend heavily on glycolysis for their energy needs. Kim et al. (2022) showed that brain endothelial cells are highly glycolytic, and that disruption of glycolysis alters BBB function [59]. Lee et al. (2022) showed that endothelial glycolysis produces lactate that supports pericyte function and helps maintain BBB integrity [60,61]. Therefore, the reduction in ECAR after SRC-2 knockdown indicates reduced glycolytic activity in brain endothelial cells. Because OCR did not increase after SRC-2 knockdown, the cells did not compensate for the reduction in glycolysis by increasing mitochondrial respiration. Instead, both OCR and ECAR decreased, suggesting that SRC-2 knockdown reduced overall cell energy metabolism.

The NAD^+^/NADH results showed that the metabolic effects of SRC-2 differ between neurons and brain endothelial cells. ps19 neurons had a lower NAD^+^/NADH ratio than WT neurons, but SRC-2 knockdown did not significantly change this ratio in neurons despite the reduction in OCR. In contrast, SRC-2 knockdown significantly reduced the NAD^+^/NADH ratio in brain endothelial cells. Together with the decrease in ECAR, this result shows that SRC-2 knockdown alters both energy metabolism and the cellular NAD^+^/NADH balance in endothelial cells.

Together, these findings provide support for a significant role of SRC-2 in vascular and metabolic regulation in the context of tauopathy. Reducing SRC-2 recapitulated several ps19-associated changes, including reduced endothelial markers, increased inhibitory eNOS phosphorylation, and reduced neuronal respiration. SRC-2 knockdown also decreased glycolytic activity and changed the NAD^+^/NADH ratio in brain endothelial cells. Not all measurements changed in the same direction as those from ps19 cortices, which is reasonable as SRC-2 regulates specific cellular processes, which may not affect all the pathways affected by tau pathology. Collectively, the data suggest that decreased SRC-2 expression contributes to the vascular and metabolic abnormalities associated with ps19 tauopathy.

## 4. Limitations and Future Directions

This study has several limitations. First, most measurements in ps19 mice were performed using whole cortical tissue, making it difficult to determine which cell types contributed to the observed changes in SRC-2, tight junction proteins, HIF-1α, and glucose transporters. Future studies using isolated brain microvessels or cell-specific SRC-2 knockout mice could help clarify these findings. Second, BBB permeability, cerebral blood flow, tissue oxygenation, and nitric oxide levels were not directly measured in vivo. Therefore, although changes in tight junction proteins and eNOS signaling suggest altered vascular regulation, their effects on BBB integrity and cerebral blood flow remain unclear. Third, some changes observed following SRC-2 knockdown in cultured brain endothelial cells differed from those measured in ps19 cortical tissue. These differences indicate that other cell types and processes associated with tau pathology may also contribute to the observed changes. In addition, examining ps19 mice at a single age does not establish how SRC-2 expression changes during disease progression. Finally, the neuronal Seahorse experiments showed increased OCR following scrambled siRNA treatment and a weak response to oligomycin. These findings complicate the interpretation of SRC-2 knockdown effects and limit the assessment of ATP-linked respiration and proton leak. Alternative knockdown approaches (e.g., viral delivery) may help address these limitations. Overall, further studies are needed to determine how SRC-2 contributes to vascular and metabolic changes in neurodegeneration and whether restoring its expression can improve these functions.

## 5. Materials and Methods

### 5.1. Transgenic Mouse Model and Experimental Design

ps19 transgenic mice, which overexpress the human 0N4R tau isoform carrying the P301S mutation, were used as the animal model in this study. Hemizygous ps19 mice [Tg(Prnp-MAPT*P301S)PS19Vle] were obtained from The Jackson Laboratory (Bar Harbor, ME, USA). ps19 transgenic mice were compared to age- and sex-matched non-transgenic littermate controls (WT). Mice were housed under controlled environmental conditions at a temperature of 22 ± 2 °C and relative humidity of 40–60%, with a 12-h light/12-h dark cycle. Food and water were provided ad libitum. Mice from both groups were euthanized at 9 months of age, and brain tissues were collected for subsequent analysis. A total of 13 mice were used in this study, including 6 wild-type (WT) and 7 ps19 mice. For immunofluorescence analysis, some tissue sections were excluded because they were damaged during preparation and unsuitable for staining. The number of animals included in each analysis is provided in the corresponding figure legends.

### 5.2. Tissue Processing and Sectioning

Whole intact brains from end-stage ps19 mice and age-matched controls were dissected, cut in half sagittally, and one half was immediately fixed in a 4% paraformaldehyde solution for 24 h. Following fixation, the brains were transferred to 70% ethanol. Subsequently, they were dehydrated, embedded in paraffin, and sliced into sections with a thickness of 6 μm and an area of 1.4 mm^2^, ensuring consistent and standardized sectioning across all samples. The other half was used to cut cortex sections, which were immediately frozen on dry ice and stored at −80 °C until use.

### 5.3. Immunofluorescence Staining

Immunofluorescence staining was performed to measure the expression levels of key proteins within the brain cortex. Primary antibodies targeting SRC-1 (ThermoFisher, Waltham, MA, USA; catalog no: PA1-840), SRC-2 (ThermoFisher, Woburn, MA, USA; catalog no: BS-3561R), SRC-3 (Cell signaling, Danvers, MA, USA; catalog no: 5765S), CD31 (abcam, Cambridge, UK; catalog no: ab222783), DyLight 594-conjugated Lycopersicon esculentum lectin (Vector Laboratories, Newark, CA, USA; catalog no. DL-1177-1), and HIF1α (Cell signaling, Danvers, MA, USA; catalog no: 36169S) were used. Fluorescent-conjugated secondary antibodies were employed to detect the primary antibodies, and all secondary antibodies were diluted in phosphate-buffered saline at a concentration of 1:1000. Fluorescent images of brain cortex sections were acquired using a confocal microscope (Nikon A1R-s with NIS-Elements software, Nikon Instruments Inc., Melville, NY, USA) at 63× magnification for CD31, lectin and HIF1α, and 40× for SRC-1, SRC-2 and SRC-3. For each mouse, three brain sections were used, and each data point in the graph represents the average of these three sections. The same parameters for acquiring images were used for all brain sections. For quantitative analysis, all intensities were normalized to their respective DAPI channel intensities (except CD31 and lectin), allowing for comparison even when acquisition parameters varied. Signal intensity analysis was conducted using ImageJ software (version 1.54p; National Institutes of Health, Bethesda, MD, USA). The normalized data were then analyzed statistically using GraphPad Prism (version 10; GraphPad Software, Boston, MA, USA)to determine if there are any significant differences between experimental groups.

### 5.4. Western Blot

Brain tissues were lysed with radioimmunoprecipitation assay buffer containing protease inhibitors. Protein concentration and purity were determined using the Bio-Rad Protein Assay Dye Reagent Concentrate (Bio-Rad Laboratories, Hercules, CA, USA; catalog no. 500-0006). The supernatant was collected after centrifugation at 15,000 rpm for 15 min. Proteins (50 μg) were mixed with 4x Laemmli sample buffer and boiled for 5 min at 95 °C. Protein samples were separated by SDS-PAGE and transferred onto a 0.2 µm PVDF membrane using the Trans-Blot Turbo Transfer System (Bio-Rad) according to the manufacturer’s instructions. The membranes were blocked with blocking buffer (2.5% non-fat dry milk in 1× TBST) for 2 h at room temperature. Primary antibodies were diluted in blocking buffer and incubated with the membranes overnight at 4 °C. On the next day, after washing, the membranes were incubated with HRP-conjugated secondary antibodies diluted in blocking buffer for 2 h at room temperature. Protein bands were visualized using an appropriate chemiluminescent substrate and captured using a Bio-Rad ChemiDoc MP Imaging System with Image Lab Touch Software (version 3.0.1; Bio-Rad Laboratories, Hercules, CA, USA).

### 5.5. siRNA-Mediated Knockdown of SRC-2 in Brain Endothelial Cells and Neurons

Brain endothelial cells (ATCC, Manassas, VA, USA; catalog no: CRL-3245) and primary neurons (isolated from mouse brain) were transfected with small interfering RNA (siRNA) targeting SRC-2 or with a non-targeting scrambled siRNA used as a negative control. A total of 5000 cells/well were seeded and allowed to adhere before transfection. SRC-2 siRNA or scrambled siRNA was introduced using an siRNA transfection reagent according to the manufacturer’s instructions. SRC-2. After a 48hr incubation period, the cells were harvested or used for further studies. SRC-2 knockdown efficiency was confirmed by measuring SRC-2 expression by qPCR.

### 5.6. Nitric Oxide Measurement

The nitric oxide-sensitive fluorescent probe DAF-FM DA (MedChemExpress, Monmouth Junction, NJ, USA; catalog no: HY-D0717) was used to measure cellular nitric oxide levels. Brain endothelial cells were seeded in 24-well plates at 2 × 10^5^ cells per well and cultured in DMEM medium (ATCC, catalog no: 30-2006) supplemented with 10% fetal bovine serum, 5% penicillin, and 40 µg/mL endothelial cell growth supplement (Millipore Sigma, Burlington, MA, USA; catalog no: 02-102) at 37 °C in a 5% CO_2_ atmosphere. At 48 h after SRC-2 siRNA or scrambled siRNA transfection, as described above, the cells were incubated with DAF-FM DA (5 μM) at 37 °C for 30 min and subsequently counterstained with DAPI. Cellular fluorescence intensity was measured using a Nikon A1 confocal microscopecs.

### 5.7. qPCR

The brain cortex was collected from the mice as described above. Total RNA was extracted using the RNeasy Maxi Kit (Qiagen, Hilden, Germany; catalog no: 74104) and its purity was determined by measuring absorbance at 260/280 nm. The RNA was used to generate complimentary DNA (cDNA) using SuperScript™ VILO™ Master Mix (ThermoFisher Scientific, Waltham, MA, USA; catalog no: 11755050). qPCR was performed using SYBR™ Green Universal Master Mix (ThermoFisher, catalog no: 4309155) and with the respective primers. GAPDH was used as an internal control in all qPCR experiments. Samples were normalized first to GAPDH and then to control samples using the ΔΔCT method. The fold change values were plotted using GraphPad Prism.

### 5.8. Neuron Isolation

Primary neurons were isolated from WT and ps19 mouse brains (age: 9 months) using the Adult Neuron Isolation Kit (Miltenyi Biotec, Bergisch Gladbach, Germany; catalog no: 130-126-603) according to the manufacturer’s protocol. Briefly, brains were dissociated into a single-cell suspension using the Adult Brain Dissociation Kit and gentle mechanical dissociation. Non-neuronal cells were magnetically labeled with a biotin-conjugated antibody cocktail targeting non-neuronal cell populations, followed by incubation with anti-biotin MicroBeads. Magnetically labeled non-neuronal cells were depleted using LS MACS columns placed in a magnetic field, and the unlabeled flow-through containing unlabeled neurons was collected. Isolated neurons were washed, counted, and used for further experiments.

### 5.9. NAD/NADH Ratio

Primary neurons isolated from 9-month-old ps19 and WT mice and brain endothelial cells were plated in a 96-well plate (5000 cells/well). The plates were taken out of the 37 °C incubator and equilibrated at room temperature for 5 min. The assay was performed directly in complete culture medium using the NAD^+^/NADH-Glo™ Assay kit (Promega, Corporation, Madison, WI, USA; catalog no: G9072). The NAD^+^/NADH-Glo™ detection reagent was prepared by combining reconstituted Luciferin Detection Reagent (1 mL) with reductase (5 µL), reductase substrate (5 µL), NAD cycling enzyme (5 µL), and NAD cycling substrate (25 µL), as per the manufacturer’s instructions (provided with the kit). NAD^+^/NADH-Glo™ Detection Reagent (50 µL) was added to each well, and the plates were gently shaken to ensure complete mixing and cell lysis. The plates were then incubated at room temperature for 30 min to allow for signal development. Luminescence was measured using a microplate luminometer at a single optimized time point.

### 5.10. Seahorse Analysis of Mitochondrial Respiration and Glycolytic Activity

Primary neurons isolated from age-matched WT and ps19 mice and brain endothelial cells were plated in Seahorse XF cell culture microplates at a density of 5000 cells/well. SRC-2 was knocked down using the siRNA protocol described above. On the day of the assay, the culture medium was replaced with Seahorse XF assay medium (Fisher scientific, Santa Clara, CA, USA; catalog no: 10-368-0100). The cells were then incubated at 37 °C in a non-CO_2_ incubator for 45–60 min before the assay. Mitochondrial respiration was measured as the OCR using the Seahorse XF Cell Mito Stress Test kit (Agilent Technologies, Santa Clara, CA, USA; part no. 103015-100) according to the manufacturer’s instructions. ECAR was measured at the same time as an indicator of glycolytic activity. After baseline oxygen consumption rate (OCR) measurements, oligomycin, FCCP, and rotenone/antimycin A were sequentially injected at final concentrations of 1.5, 0.5, and 0.5 µM, respectively, for BECs, and 1, 0.5, and 1 µM, respectively, for primary neurons. Basal respiration was determined from OCR measurements obtained before oligomycin treatment. The change in OCR following oligomycin treatment was used to assess ATP-linked respiration, while the response to FCCP was used to evaluate maximal mitochondrial respiration. The OCR after rotenone/antimycin A treatment was used to measure non-mitochondrial oxygen consumption in neurons and brain endothelial cells.

## Data Availability

The raw data supporting the conclusions of this article will be made available by the authors, without undue reservation.

## Supplementary Materials

The following supporting information can be downloaded at: https://www.mdpi.com/article/10.3390/ijms27219943/s1.
