# Supplementary material for: SRC-2 Regulates Brain Endothelial and Neuronal Metabolism in a Mouse Model of Neurodegeneration
Source: Int J Mol Sci. 2026 Nov 6;27(21):9943. doi: 10.3390/ijms27219943 (PMC13764533; doi:10.3390/ijms27219943)
Supplement: Supplementary file 1 [file ijms-27-09943-s001.zip › ijms-4670058-supplementary.pdf]

Supplementary Figures

Supplementary Figure S1:

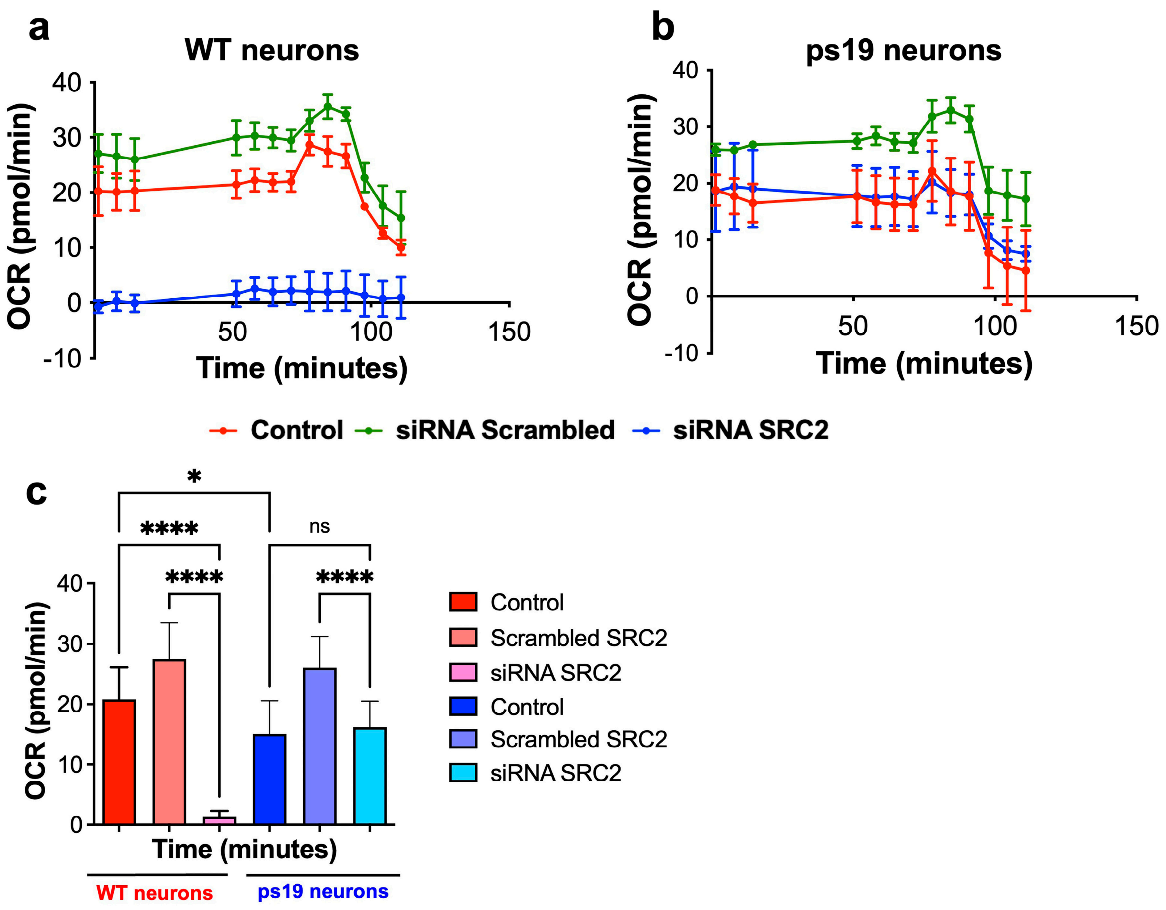

Supplementary Figure S1. SRC-2 knockdown reduces mitochondrial respiration in primary neurons isolated from WT and ps19 mice. (a) Oxygen consumption rate (OCR) in primary neurons isolated from wild-type (WT) mice following no treatment (control), scrambled siRNA treatment, or SRC-2 siRNA-mediated knockdown, measured using the Seahorse extracellular flux analyzer. (b) OCR in primary neurons isolated from ps19 mice under the same experimental conditions. The line graphs show OCR over time following sequential injection of mitochondrial stress test reagents. (c) Quantification of basal OCR in untreated control, scrambled siRNA-treated, and SRC-2 siRNA-treated neurons from WT and ps19 mice.

Supplementary Figure S2:

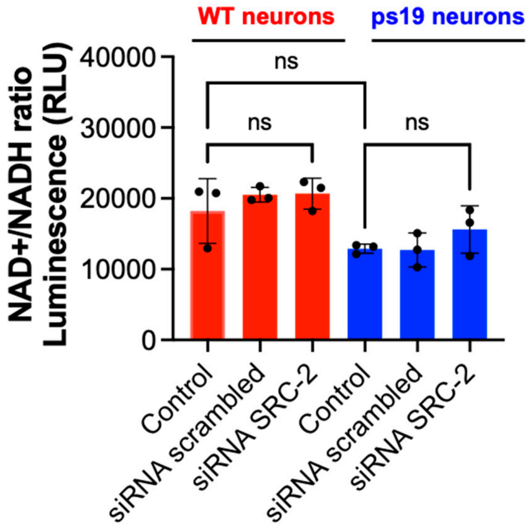

<u>Supplementary Figure S2</u>. NAD$^+$/NADH ratio in primary neurons isolated from WT and ps19 mice following SRC-2 knockdown. NAD$^+$/NADH ratios were measured in primary neurons isolated from wild-type (WT) and ps19 mice under untreated control, scrambled siRNA-treated, and SRC-2 siRNA-treated conditions. The NAD$^+$/NADH ratio was lower in ps19 neurons than in WT neurons. SRC-2 knockdown did not significantly alter the NAD$^+$/NADH ratio in either WT or ps19 neurons compared with their respective scrambled siRNA controls.
